# Human immune reactivity of *GGTA1/CMAH/A3GALT2* triple knockout Yucatan miniature pigs

**DOI:** 10.1007/s11248-021-00271-w

**Published:** 2021-07-07

**Authors:** Joohyun Shim, Nayoung Ko, Hyoung-Joo Kim, Yongjin Lee, Jeong-Woong Lee, Dong-Il Jin, Hyunil Kim, Kimyung Choi

**Affiliations:** 1 Department of Transgenic Animal Research, Optipharm, Inc., Chungcheongbuk-do, Cheongju-si, 28158 Republic of Korea; 2 Biotherapeutics Translational Research Center, Korea Research Institute of Bioscience and Biotechnology, Daejeon, Republic of Korea; 3 Department of Animal Science and Biotechnology, Chungnam National University, Daejeon, Republic of Korea

**Keywords:** CRISPR/Cas9, *GGTA1*, *CMAH*, *A3GALT2*, Antibody binding, Cytotoxicity

## Abstract

In this study, we investigated the effect of a triple knockout of the genes alpha-1,3-galactosyltransferase (*GGTA1*), cytidine monophosphate-N-acetylneuraminic acid hydroxylase (*CMAH*), and alpha 1,3-galactosyltransferase 2 (*A3GALT2*) in Yucatan miniature pigs on human immune reactivity. We used the CRISPR/Cas9 system to create pigs lacking *GGTA1* (GTKO) and *GGTA1/CMAH/A3GALT2* triple gene knockout (TKO). The expression of all three xenoantigens was absent in TKO pigs, but there was no additional reduction in the level of Galα1,3Gal (αGal) epitopes expression in the *A3GALT2* gene KO. Peripheral blood mononuclear cells (PBMCs), aorta endothelial cells (AECs), and cornea endothelial cells (CECs) were isolated from these pigs, and their ability to bind human IgM/IgG and their cytotoxicity in human sera were evaluated. Compared to wild type (WT) pigs, the level of human antibody binding of the PBMCs, AECs, and CECs of the transgenic pigs (GTKO and TKO) was significantly reduced. However, there were significant differences in human antibody binding between GTKO and TKO depending on the cell type. Human antibody binding of TKO pigs was less than that of GTKO on PBMCs but was similar between GTKO and TKO pigs for AECs and CECs. Cytotoxicity of transgenic pig (GTKO and TKO) PBMCs and AECs was significantly reduced compared to that of WT pigs. However, TKO pigs showed a reduction in cytotoxicity compared to GTKO pigs on PBMCs, whereas in AECs from both TKO and GTKO pigs, there was no difference. The cytotoxicity of transgenic pig CECs was significantly decreased from that of WT at 300 min, but there was no significant reduction in TKO pigs from GTKO. Our results indicate that genetic modification of donor pigs for xenotransplantation should be tailored to the target organ and silencing of additional genes such as *CMAH* or *A3GALT2* based on GTKO might not be essential in Yucatan miniature pigs.

**Supplementary Information:**

The online version contains supplementary material available at 10.1007/s11248-021-00271-w.

## Introduction

Pigs are similar to humans with respect to their genetics, anatomy, and physiology, making them the best donor of biological materials for xenotransplantation (Butler et al. 2015; Cooper et al. 2017; Hryhorowicz et al. 2017). Despite this similarity, there is a considerable phylogenetic distance between pigs and humans, which can cause problems with the immune system following xenotransplantation (Hryhorowicz et al. 2017). To facilitate successful xenotransplantation using pig organs and tissues, the generation and characterization of transgenic pigs with reduced immunogenic properties are important (Niemann and Petersen 2016).

In pig-to-human xenotransplantation, Galα1,3Gal (αGal) is a major carbohydrate causing hyperacute rejections. Anti-Gal antibodies in human blood recognize the Gal antigen expressed in pig cells and activate a complement cascade, which eventually leads to cell lysis (Hryhorowicz et al. 2017; Niemann and Petersen 2016; Song and Kim 2013). Therefore, the removal of the αGal antigen from xenograft cell surfaces helps in producing transplantable organs. N-glycolylneuraminic acid (Neu5Gc), another carbohydrate xenoantigen, has been identified as another key antigen that is recognized by human antibodies and causes organ rejection (Wang et al. 2014). Neu5Gc is synthesized from *N*-acetylneuraminic acid (Neu5Ac) by cytidine monophosphate-*N*-acetylneuraminic acid hydroxylase (*CMAH*). It is expressed in the tissues of most mammals, including pigs and monkeys, but humans have an inactivated *CMAH* gene, so only Neu5Ac is present on human cells (Breimer 2011). Previous reports have suggested that *GGTA1* gene knockout (GTKO) pigs had an increased expression of sialyltransferase-related genes and thus increased production of Neu5Gc. These findings suggest that *CMAH* gene knockout (KO) is necessary to prevent further rejection of xenografts (Park et al. 2012; Park et al. 2011). Kwon et al. generated biallelic *CMAH* KO pigs, and showed that the transcription level of non-Gal antigen-related genes decreased in *CMAH* KO pig cells (Kwon et al. 2013). Many studies have reported that *GGTA1/CMAH* double KO pig cells had significantly less antigenicity than GTKO pig cells (Li et al. 2015; Li et al. 2013; Lutz et al. 2013; Sato et al. 2014; Whitworth et al. 2014). Butler et al. reported that *GGTA1/CMAH* double KO pig livers consumed fewer human platelets in a liver perfusion model (Butler et al. 2016a). In recent years, genome engineering techniques, including zinc-finger nucleases (ZFNs), transcription activator-like effector nucleases (TALENs), and clustered regularly interspaced short palindromic repeats (CRISPR/Cas9), have been used to produce precisely targeted genetic modifications in pigs (Butler et al. 2015; Hai et al. 2014; Ryu et al. 2018; Zhang et al. 2017b). Estrada et al. created *GGTA1/CMAH/β4GalNT2* triple KO pigs using CRISPR/Cas9, and cells from these transgenic pigs had reduced xenoreactive antibody binding in peripheral blood mononuclear cells (PBMCs). These authors suggest that the reduction of xenoantigens through the deletion of *GGTA1/CMAH/β4GalNT2* might reduce the antibody barrier and increase the success of xenotransplantation (Estrada et al. 2015). With the development of gene editing technology, pig-to-nonhuman primate xenotransplantation studies have made considerable progress. Researchers have successfully transplanted kidneys from Gal and Sda double xenoantigen KO pigs into rhesus monkeys, with the transplants functioning for 435 days (Adams et al. 2018). *GTKO/hCD46/hTBM* pig hearts were heterotopically transplanted into baboons that then survived for 945 days (Mohiuddin et al. 2016). Längin et al. extended the survival of baboons receiving life-supporting heart transplants by up to 195 days using an optimized process to preserve pig hearts during transplantation (Langin et al. 2018).

Several researchers have reported that a residual amount of αGal epitope reactivity has been found in GTKO mice tissues and GTKO pig cells (Milland et al. 2006; Milland et al. 2005; Sharma et al. 2003), suggesting that this epitope might be synthesized by other galactotransferase enzyme family proteins (Butler et al. 2016b; Kuwaki et al. 2005; Sharma et al. 2003; Yamada et al. 2005). The Gal antigen is synthesized by glycoprotein *GGTA1* or glycosphingolipids alpha 1,3-galactosyltransferase 2 (*A3GALT2, also known as iGb3s*). As with *GGTA1* and *CMAH* genes, humans possess the *A3GALT2* gene, but it is thought to be inactive owing to several mutations (Christiansen et al. 2008). Some studies have suggested that isoglobotriosylceramide (iGb3, synthesized by A3GALT2) could be another source of αGal and that the *A3GALT2* gene must also be deleted for complete removal of αGal (Christiansen et al. 2008; Milland et al. 2005). On the other hand, recent studies have shown the evidence that *A3GALT2* is unlikely to be a xenoantigen for xenotransplantation (Butler et al. 2016b; Sanderson et al. 2013; Tahiri et al. 2013). Shao et al. suggested that the *A3GALT2* gene can affect the expression of the Gal epitope, but the effect on its immunological properties in *A3GALT2* KO mice may be very weak (Shao et al. 2018). Butler et al. showed that the *A3GALT2* gene could change the renal glycosphingolipid profile but had no effect on antibody binding, or the cytotoxicity of porcine PBMCs in baboon and human sera (Butler et al. 2016b). However, iGb3 has been identified as an endogenous ligand, which is recognized by the invariant natural killer T (iNKT) cells in mice and human (Mattner et al. 2005; Zhou et al. 2004). In addition, it has been suggested that the expression of iGb3 on pig cells may trigger the activation of natural killer T (NKT) cells on adaptive immune cells (Christiansen et al. 2008). As the role of iGb3 on the development and function of NKT cells is still not clear, a study of *A3GALT2* KO in pigs is required.

In this study, we generated *GGTA1/CMAH/A3GALT2* triple knockout (TKO; TKO pigs mean silencing a gene not *β4galNT2* but *A3GALT2* along with *GGTA1* and *CMAH*) pigs using CRISPR/Cas9 and examined the effect of the elimination of these genes on pig-to-human immune reactivity.

## Materials and methods

### Animals and chemicals

All animal care and use procedure protocols were approved by the Institutional Animal Care and Use Committee of Optipharm, Inc., Life Science Institute (IACUC approval No. OPT-140103-1). All pigs were housed in the animal facility at Optipharm, Inc., Korea, in a specific pathogen-free environment. The room was maintained at 24 °C ± 2 °C and 12 h light/12 h dark cycles. Filtered air and water, and sterilized feed were supplied. All pigs in this study were blood type O Yucatan miniature pigs, except for the surrogate gilts (Landrace/Yorkshire/Duroc cross-breed). For further experiments, six- to seven-week-old healthy pigs, GTKO, TKO, and wild type (WT) were humanely euthanized, and their organs harvested. The euthanasia was performed by intravenous injection of 2 mmol/kg potassium chloride solution under general anesthesia, and a veterinarian certified the death of the animals. Unless otherwise noted, all chemicals were purchased from Sigma-Aldrich (St. Louis, MO, USA).

### Establishment of transgenic cell lines

For the establishment of the GTKO cell lines, a pair of oligos specific for the targeted site of *GGTA1* (5′-GCAAATACATACTTCATGGT-3′) were hybridized using a thermal cycler. Ear fibroblast cells derived from Yucatan miniature pigs were transfected with Cas9-GFP-GGTA1 plasmids using electroporation (Amaxa 4D, Lonza, Walkersville, MD, USA). After 48 h, transfected cells were selected using FACS AriaII (BD Bioscience, San Diego, CA, USA) and seeded on a 10 cm culture dish. Single cell clusters were transferred to 96-well plates, and serial amplification was performed on colonies from the 10 cm culture dish. To establish the TKO cell lines, the guide RNA sequences for *CMAH* (5′-AACTCCTGAACTACAAGGCT-3′) and *A3GALT2* (5′-GACGTGGATCAGCACTTCAG-3′) were propagated. GTKO cells were transfected with both Cas9-GFP-CMAH and Cas9-GFP-A3GALT2 vectors to establish the TKO cell lines using Lipofectamine 3000 (Invitrogen, San Diego, CA, USA) according to the manufacturer’s protocol. All cell lines were then produced using the procedure described below.

### Analysis of CRISPR/Cas9-induced mutations in nuclear donor cells and cloned piglets

Genomic DNA from transgenic cells and cloned piglets were extracted using DNeasy extraction kits (Qiagen, Hilden, Germany). PCR was performed using *GGTA1*-, *CMAH*-, and *A3GALT2*-specific primer sets (S1 Table). The primer sets were designed to include the target sites and produced amplicons of 496 base pairs for *GGTA1*, 328 base pairs for *CMAH*, and 344 base pairs for *A3GALT2*. The PCR conditions for *GGTA1* were as follows: initial denaturation step of 95 °C for 5 min; 95 °C for 30 s, 61 °C for 30 s, and 72 °C for 30 s; for 35 cycles; and final extension at 72 °C for 5 min. The PCR conditions for *CMAH* were as follows: initial denaturation step of 95 °C for 5 min; 95 °C for 30 s, 52 °C for 30 s, and 72 °C 30 s; for 35 cycles; and final extension at 72 °C for 5 min. For *A3GALT2*, the PCR conditions were as follows: initial denaturation step of 95 °C for 5 min; 95 °C for 30 s, 60 °C for 30 s, and 72 °C 30 s; for 35 cycles; and final extension at 72 °C for 5 min. The PCR products were confirmed by sequencing (Solgent, Daejeon, Korea).

### Somatic cell nuclear transfer (SCNT) and embryo transfer (ET)

SCNT was performed as described in our previous study (Choi et al. 2017). Ovaries were obtained from a local slaughterhouse (Farmstory Hannaeng, Cheongju, Korea). Immature oocytes were cultured in M199 (Gibco, Waltham, MA, USA) based maturation medium for 42–44 h at 38.5 °C under 5% CO_2_ in air. Matured oocytes at the MII stage were enucleated by aspirating the first polar body, chromosomes, and the adjacent cytoplasm with a fine glass pipette in M199 medium (supplemented with 0.3% bovine serum albumin (BSA) and 7.5 μg/mL cytochalasin B). A single donor cell at the G0/G1 stage of the cell cycle was injected into the perivitelline space. The reconstructed oocytes were fused in 0.3 M mannitol medium (BTX, Holliston, MA, USA). Fused embryos were cultured in porcine zygote medium (PZM-3) supplemented with 0.4% BSA. Embryos were surgically transferred to oviducts of a surrogate gilt on day 0 or 1 of estrus. All surrogate gilts were anesthetized with an intravenous injection of Alfaxan (0.5 mg/kg; Jurox Pty. Ltd., NSW, Australia) and Domitor (10 μg/kg; Orion Pharma, Berkshire, UK), and anesthesia was maintained using inhaled isoflurane (Hana Pharm, Hwasung, Korea) at concentrations of <  5%. Pregnancy was monitored at day 28 after ET using an ultrasound scanner (PA60A; Samsung Medison, Seoul, Korea). The cloned piglets were delivered by natural birth.

### Culture of porcine AECs and CECs

Porcine AECs and CECs were isolated, as previously described, with slight modifications (Kim et al. 2016; Lee et al. 2016a). Porcine aortas were digested with collagenase. The cells were washed with Dulbecco’s phosphate buffered saline (DPBS, Invitrogen), and then cultured in porcine endothelial cell culture medium (Lonza, Basel, Switzerland). Corneal tissues were dissected and incubated with 0.25% trypsin-EDTA (Invitrogen) for 20 min at 37 °C. CECs were peeled off and cultured in M199 medium (Gibco) containing 10% fetal bovine serum (Hyclone, UT, USA).

### Isolation of PBMCs

Animals over 12 months old were used for blood collection. Blood from GTKO, TKO, and WT pigs was collected in EDTA tubes (BD Bioscience). PBMCs were separated from the whole blood using Ficoll-Paque PLUS (GE, UK) according to the manufacturer’s protocol. Separated PBMCs were washed with PBS (Gibco). Isolated PBMCs were then used immediately for human complement-mediated cytotoxicity or human antibody binding assays.

### Immunofluorescence assay (IFA) from organ tissues

Tissues were fixed using 4% paraformaldehyde and made into paraffin blocks. Tissue sections were used for the immunofluorescence analysis of two antigens. αGal was stained with isolectin GS-IB4, Alexa Fluor® 594 conjugate (Life Technologies, Carlsbad, CA, USA). Neu5Gc was stained with chicken anti-Neu5Gc antibody (BioLegend, San Diego, CA, USA) and goat anti-chicken fluorescein isothiocyanate (FITC) (Abcam, Cambridge, UK) following the manufacturer’s protocols. The tissues were imaged using a confocal laser scanning microscope (Zeiss, Oberkochen, Germany).

### Expression of A3GALT2 and αGal epitopes

The expression of A3GALT2 and αGal epitopes in pig cells was analyzed using FACS analysis with A3GALT2 antibody, a synthetic polyclonal antibody against pig A3GALT2 in rabbits, and anti-αGal antibody (Enzo Life Sciences, NY, USA). AECs and PBMCs were stained with mouse anti-αGal epitope antibody for 2 h at room temperature and goat anti-mouse Dylight 488 (Abcam) for 1 h on ice. AECs were stained with A3GALT2 antibody for 2 h at room temperature and goat anti-rabbit Dylight 488 (Abcam) for 1 h on ice. Flow cytometry was performed using BD FACS CantoII (BD biosciences, CA, USA), and the results were analyzed using FlowJo software (version 10; https://www.flowjo.com/).

### Antibody binding of IgM and IgG to pig cells

The binding of human antibody to pig cells was evaluated as previously described (Hara et al. 2008). Isolated PBMCs and harvested AECs and CECs were incubated in 10% normal goat serum for 30 min at 4 °C. Cells were then washed twice with DPBS. The washed cells were incubated in 10% normal human serum (NHS; EMD Millipore, Burlington, MA, USA) or DPBS (negative control) for 30 min (PBMCs) or 2 h (AECs and CECs) at 4 °C. Cells were washed with DPBS and incubated with FITC-conjugated goat anti-human IgM (μ chain-specific) and IgG (γ chain-specific, Invitrogen) antibodies for 1 h at 4 °C. Binding of human IgM and IgG to PBMCs, AECs, and CECs was detected using a flow cytometer (FACS CantoII, BD) and quantified using relative mean fluorescence intensity (MFI), calculated as follows: Relative MFI = (MFI value of target)/(MFI value of negative control).

### Human complement-mediated cytotoxicity assay

We conducted a complement-dependent cytotoxicity assay of the PBMCs, AECs, and CECs collected from TKO, GTKO, and WT pigs. Cells were counted using an EVE cell counter (Nanoentek, Seoul, Korea) and incubated in 50% pooled complement human serum (Innovative Research, Peary Court Novi, MI, USA) for 300 min. After incubation, human serum was removed by centrifugation. The cells were then resuspended in 100 μL of cell culture media. To estimate the viability of the cells, 10 μL of CCK-8 solution (Dojindo, Kumamoto, Japan) was added, and the cells were then incubated for another 2 h. Cytotoxicity was analyzed using absorbance at a wavelength of 450 nm recorded using a microplate reader (Infinite M200 pro NanoQuant, Tecan, Mannedorf, Switzerland).

### Statistical analysis

Statistical analysis was conducted using GraphPad Prism version 6 (GraphPad Software, San Diego, CA, USA). One-way or two-way (for cytotoxicity) analysis of variance (ANOVA) followed by Bonferroni post-hoc testing was used to evaluate significance.

## Results

### Generation of GTKO and TKO fibroblasts

**Fig. 1:**
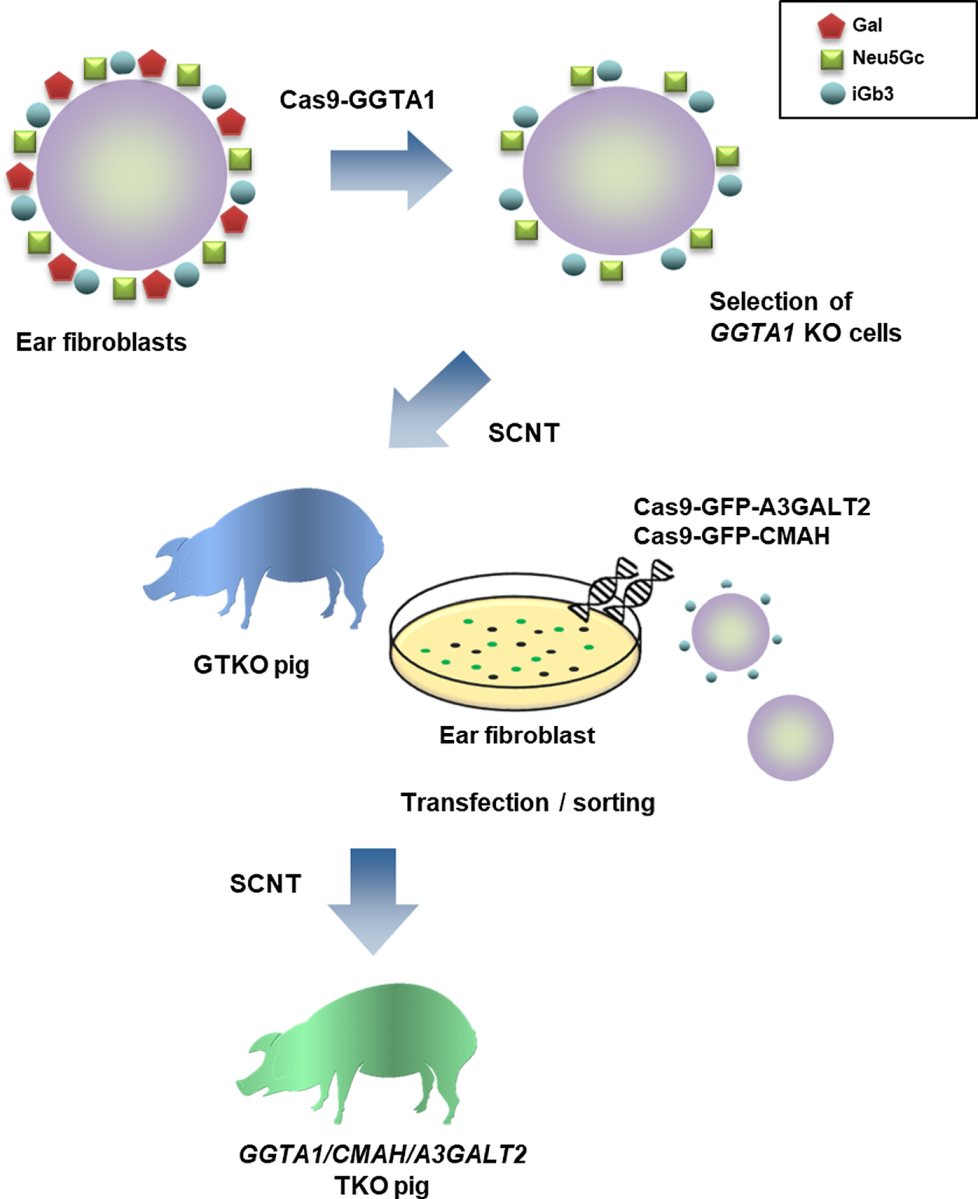
Workflow of the generation of *GGTA1*, *CMAH*, and *A3GALT2* gene KO pigs using CRISPR/Cas9 technology. Ear fibroblast cells of Yucatan miniature pigs were transfected with a Cas9-GGTA1 vector. We produced cloned *GGTA1* KO (GTKO) piglets using GS-IB4 lectin negative cells as nuclear donors for SCNT. We then simultaneously targeted the *CMAH* and *A3GALT2* genes using CRISPR/Cas9 in ear fibroblast cells from a GTKO piglet. SCNT was performed with these transfected cells, and *GGTA1/CMAH/A3GALT2* triple gene KO (TKO) piglets were produced.

**Fig. 2:**
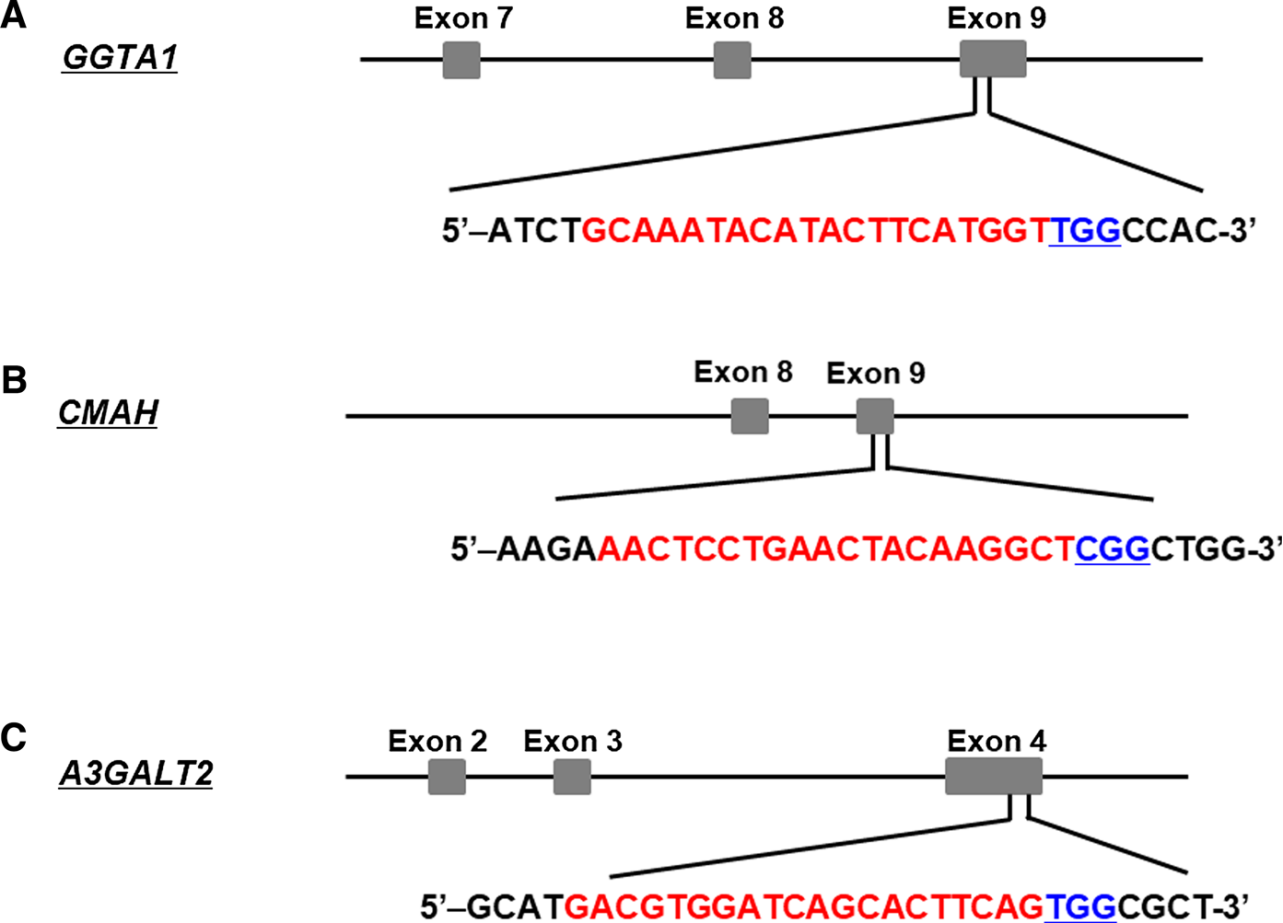
Generation of *GGTA1*, *CMAH*, and *A3GALT2* gene KO transgenic cell lines. Schematic diagram of the CRISPR/Cas9 system targeting pigs. **a** *GGTA1*, **b** *CMAH*, and **c** *A3GALT2* locus. The target site was in exon 4 of *A3GALT2* and exon 9 of *GGTA1* and *CMAH* genes. The red highlight indicates sgRNA targeting sites, and the blue underlined highlight indicates protospacer adjacent motif (PAM).

**Fig. 3:**
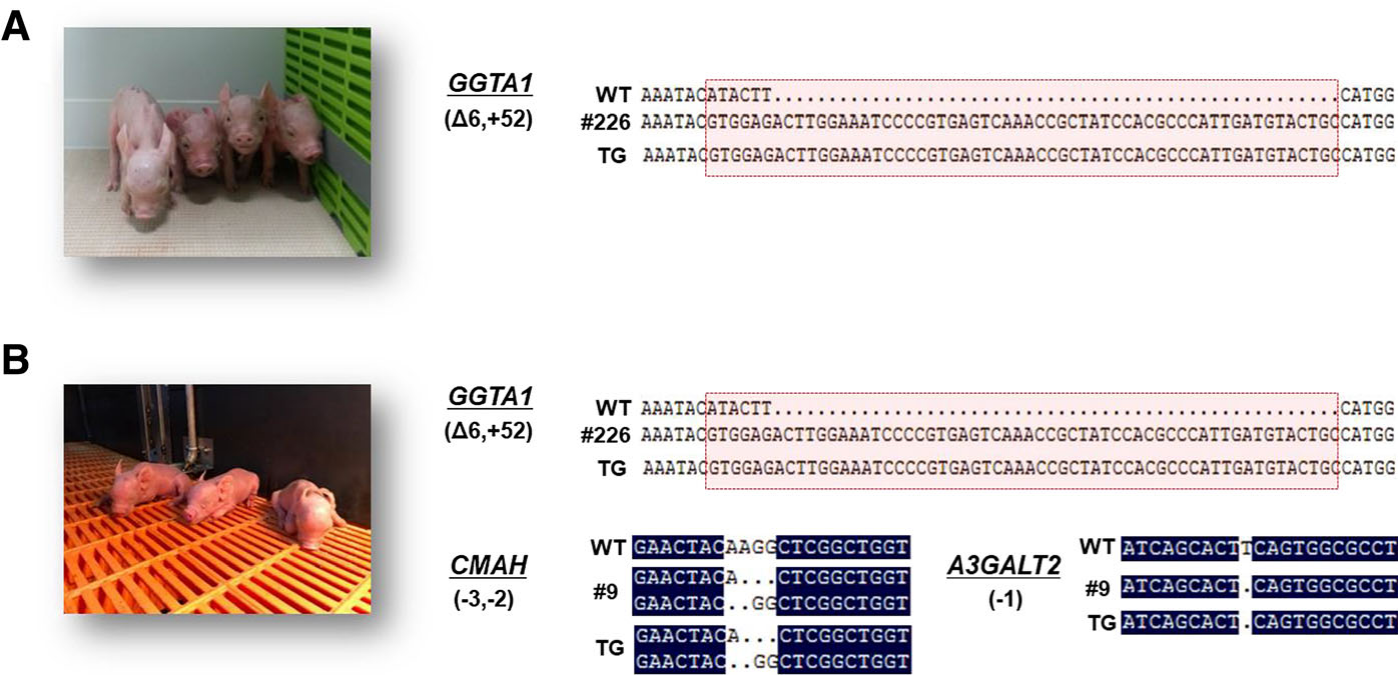
Generation of CRISPR/Cas9-induced GTKO and TKO cloned piglets. DNA sequence analysis and photographs of **a** GTKO and **b** TKO piglets. The DNA sequences of the cloned piglets showed a mutation identical to the sequences of the nuclear donor cells. Deletion, insertion, and shift mutations of the base pairs are indicated by dots (∙), plus (+), and delta (Δ), respectively.

Figure 1 outlines the process used to produce genetically modified pigs. For the *GGTA1* gene, sgRNAs were designed to target exon 9 as shown in Fig. 2a. Porcine ear fibroblast cells were transfected with the CRISPR/Cas9 vector for *GGTA1* KO, and 13 *GGTA1* mutant colonies were selected (13/21, 61.9%; Table 1 and Fig. S1a). Of these mutants, #226 was selected as the source of donor cells for SCNT using DNA sequencing (Fig. 3a). This procedure was repeated for the other two genes with sgRNAs designed to target exon 9 of *CMAH* and exon 4 of the *A3GALT2* genes (Fig. 2b, c). The ear fibroblast cells isolated from GTKO piglets were simultaneously transfected with the CRISPR/Cas9 vectors for *CMAH* and *A3GALT2* KO. Subsequently, 16 colonies carrying mutations in both *CMAH* and *A3GALT2* (16/19, 84.2%) were identified and confirmed by DNA sequencing (Table 1 and Fig. S1b, c).

**Table 1.** Targeting efficiency of CRISPR/Cas9

Target gene	Target cells	Sex	Cell cloned with mutations (%)
*GGTA1*	WT ear cells	Male	13/21 (61.9)
*CMAH/A3GALT2*	GTKO ear cells	Male	16/19 (84.2)

### Production of GTKO and TKO pigs by SCNT

A total of 401 reconstructed embryos derived from the *GGTA1* KO cells were transferred into two surrogate recipients. One surrogate became pregnant and gave birth to five piglets, of which one piglet was stillborn and four piglets were live born (Table 2). All piglets were biallelic for the *GGTA1* gene KO as determined by DNA sequencing (Fig. 3a). Flow cytometry analysis and western blot assay of the ear cells showed that the GTKO piglets did not express αGal (Fig. S2). We then attempted to generate cloned pigs using the selected TKO donor cells. A total of 718 reconstructed embryos were transferred into five recipient gilts and four became pregnant. All surrogate recipients carried to full term. Two piglets were stillborn, and eight piglets were live born (Table 2). The genotypes of the cloned piglets were determined using PCR and sequencing. All of the cloned piglets contained deletions of the *GGTA1, CMAH,* and *A3GALT2* genes like the donor cells (Fig. 3a, b).

**Table 2.** Efficiency of SCNT using transgenic donor cells

Donor cells	No. of recipients	No. of transferred embryos (embryos per transfer)	Pregnancy (%)^a^	Delivery (%)	Live born (still born)	Cloning efficiency^b^
GTKO	2	401 (200)	1 (50)	1 (50)	4 (1)	1.2
TKO	5	718 (144)	4 (80)	4 (100)	8 (2)	1.3

^a^Pregnant animals/total recipients

^b^Fetuses and piglets/total embryos transferred

### Expression of αGal, Neu5Gc and A3GALT2

**Fig. 4:**
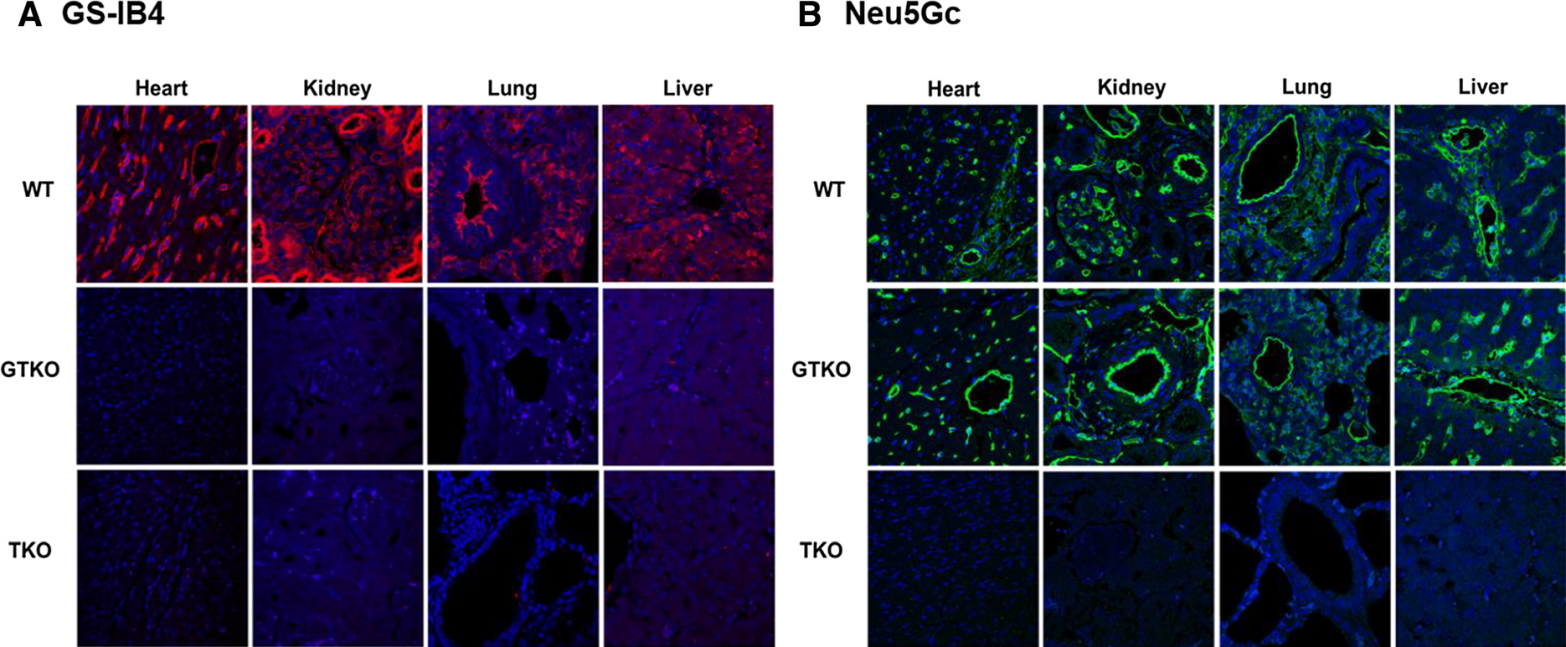
Immunofluorescence analysis of GTKO, TKO, and WT pigs. Tissue sections of the heart, kidney, lung, and liver from GTKO, TKO, and WT pigs were stained with **a** GS-IB4 lectin **b** anti-Neu5Gc antibodies. Expression of αGal and Neu5Gc antigens was widespread in WT pig tissues. All tissues from GTKO pigs were negative for αGal, but TKO pigs were negative for both αGal and Neu5Gc antigens (nuclei, blue; Gal, red; Neu5Gc, green, magnification, × 400).

**Fig. 5:**
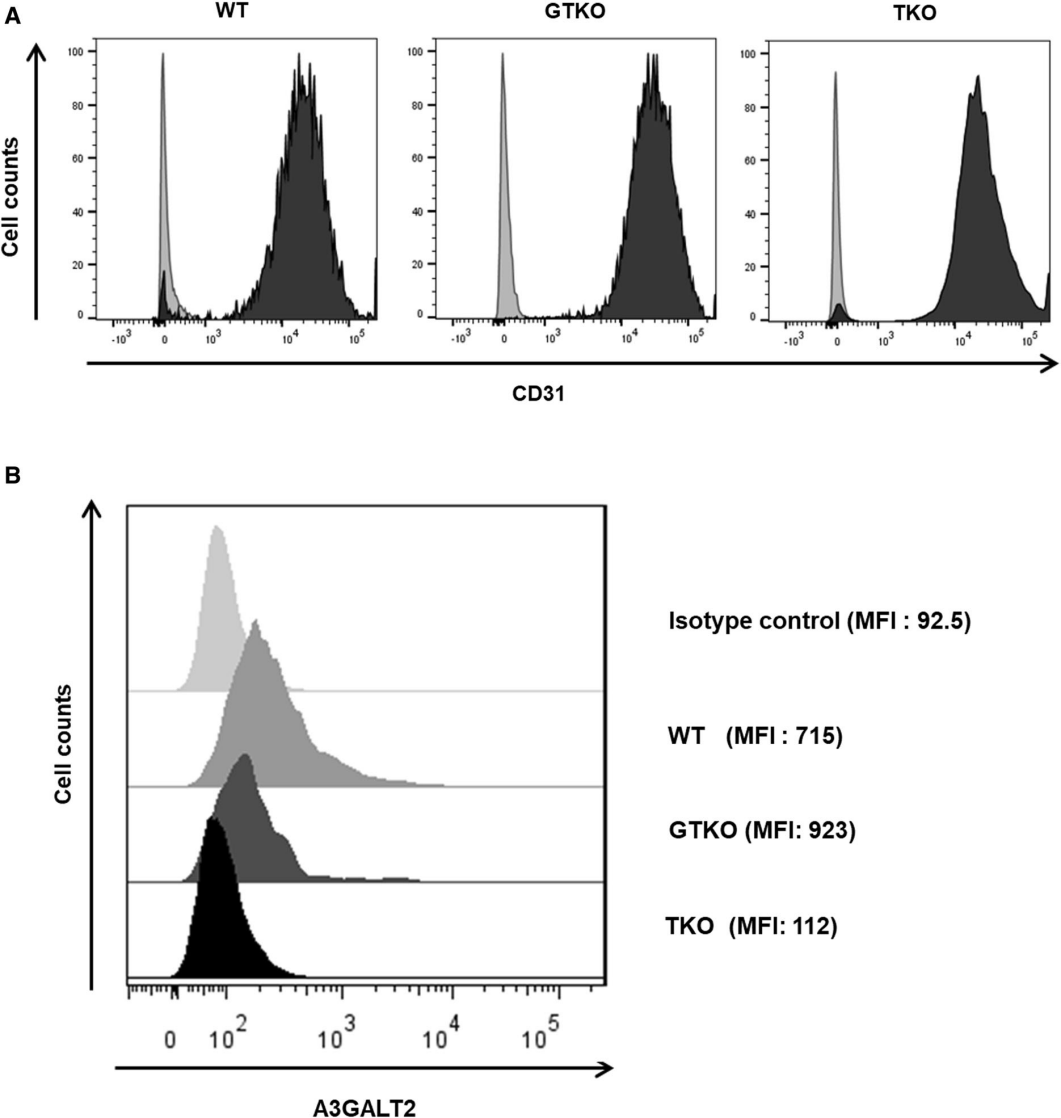
Comparison of A3GALT2 expression in AECs from GTKO, TKO, and WT pigs. **a** Endothelial cell marker CD31 was expressed on AECs from all types of pigs. Gray histograms represent isotype control, and black histograms represent CD31. **b** The expression level of A3GALT2 on TKO pig AECs was significantly decreased from that of GTKO or WT pigs (MFI; mean fluorescence intensity).

**Fig. 6:**
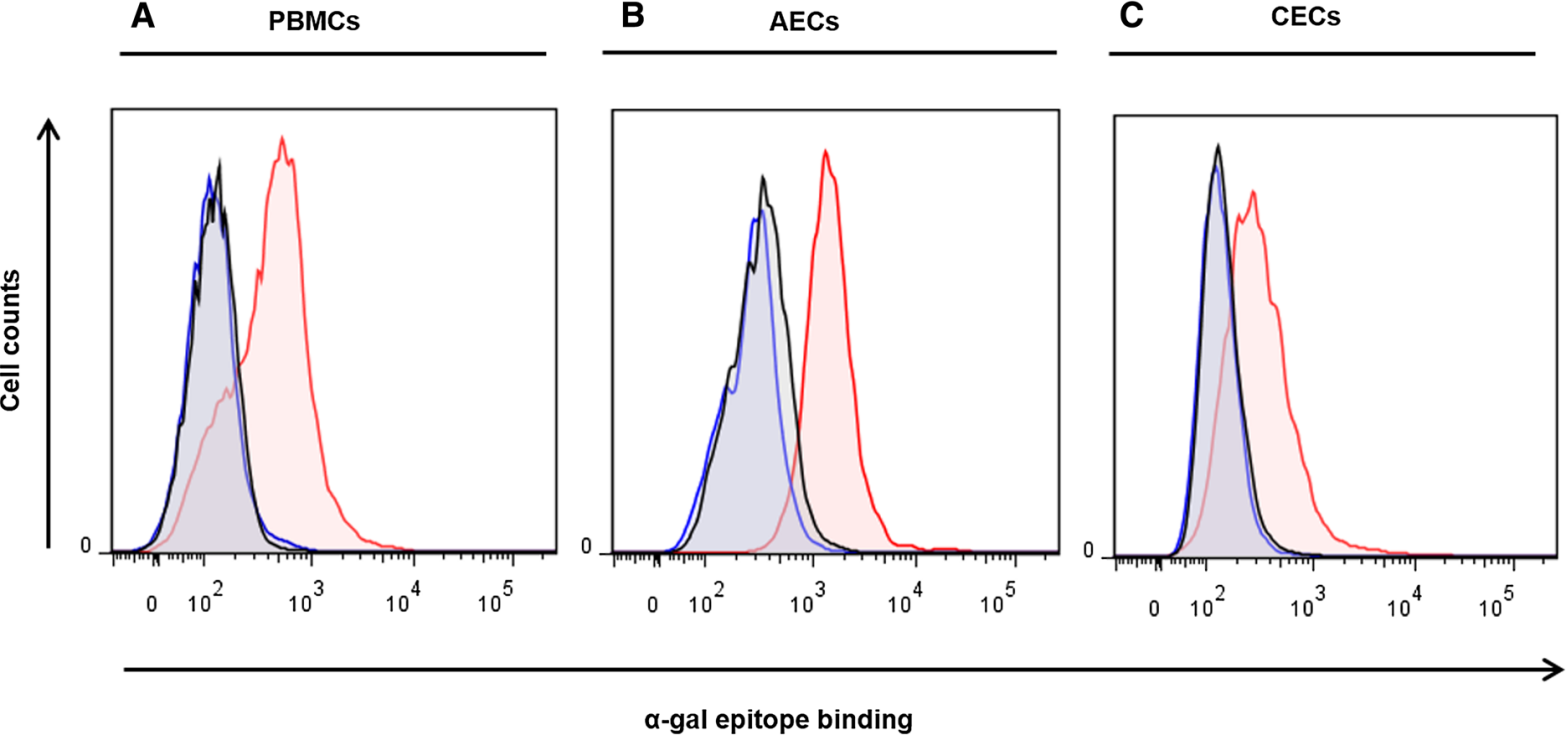
Comparison of αGal epitope expression on various pig cells. αGal epitope expression on** a** PBMCs, **b** AECs, and **c** CECs from all transgenic pigs was decreased from that of WT. However, there were no differences in the expression of αGal between GTKO and TKO pig cells (WT, red; GTKO, blue; TKO, black).

The expression of αGal and Neu5Gc antigens was examined in the heart, kidney, lung, and liver from GTKO, TKO, and WT pigs using immunofluorescence (IFA). αGal was expressed at a high level in the cardiac muscle, capillary endothelia, kidney tubules, pulmonary alveoli, and hepatocytes in a WT pig but was not detected in any of the transgenic pig tissues. However, we could not identify an additional reduction of αGal in TKO pig organ tissues when compared with GTKO pigs using the IFA assays (Fig. 4a). Neu5Gc was primarily expressed in the capillaries and vessels of all tissues in the WT and GTKO pigs (Fig. 4b). Neither αGal nor Neu5Gc was observed in any of the tissues from a TKO pig (Fig. 4a, b). We also examined the expression of A3GALT2 in various organ tissues of GTKO, TKO, and WT pigs and found no visible difference among them (data not shown). We then compared the expression of A3GALT2 from GTKO, TKO, and WT pigs at the cellular level (AECs) using FACS analysis. As shown in Fig. 5, the expression level of A3GALT2 in the TKO pig AECs was approximately eight-fold lower than that in AECs from WT or GTKO pigs (Fig. 5). To identify correlations between the level of αGal epitope expression and the silencing of the *A3GALT2* gene, we evaluated αGal expression in pig PBMCs, AECs, and CECs using anti-Gal antibody. The expression of the αGal epitope in PBMCs, AECs, and CECs in all of the transgenic pigs was decreased from that in WT pigs. There were no differences in αGal expression in any of the cell types of GTKO and TKO pigs (Fig. 6).

### Human IgM/IgG binding to pig cells

**Fig. 7:**
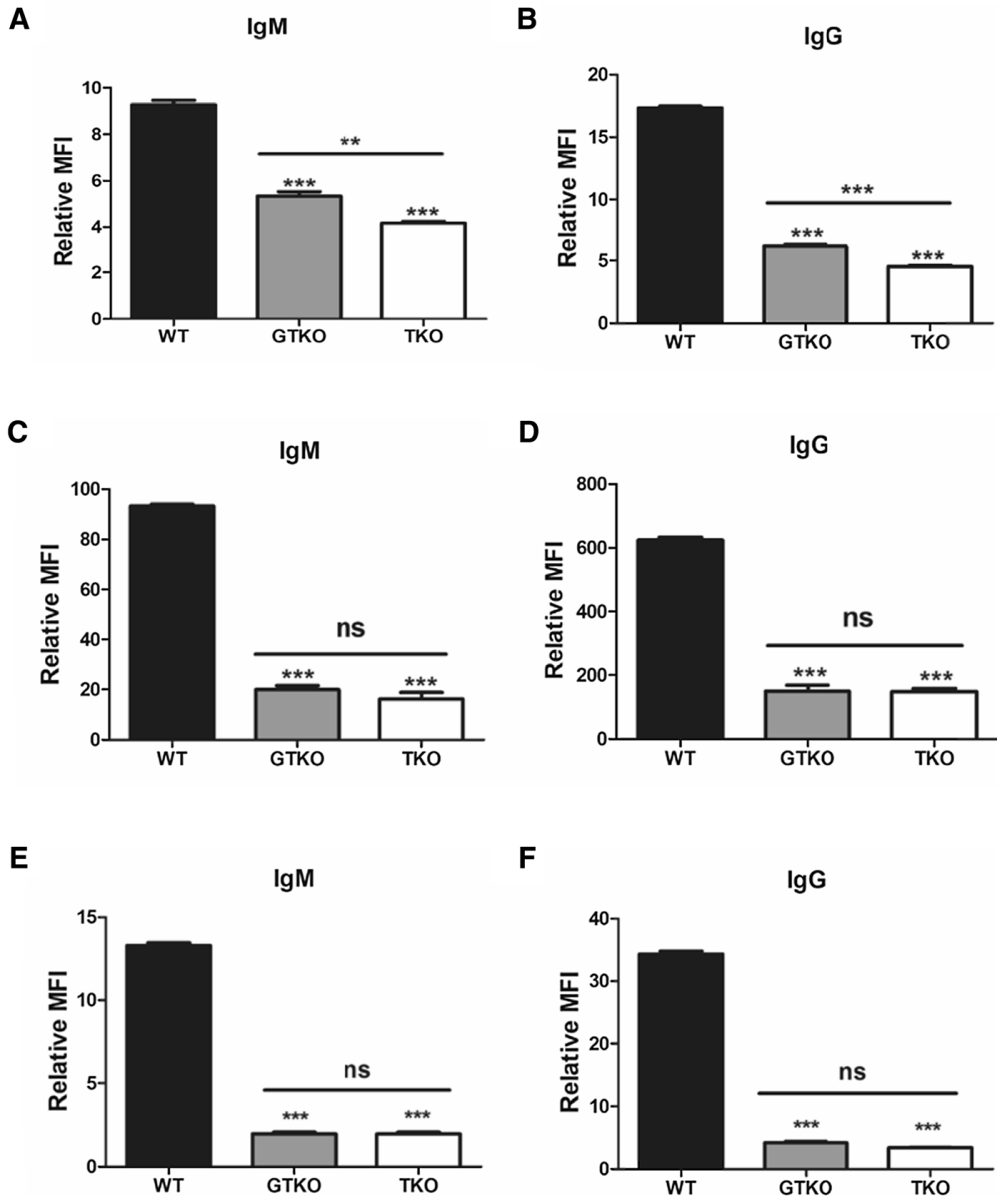
Human IgM and IgG binding to GTKO, TKO, and WT pig PBMCs (**a, b**), AECs (**c, d**), and CECs (**e, f**). **a**, **b** Human IgM/IgG binding of PBMCs was significantly decreased in transgenic pigs when compared to WT (***P < 0.001 vs. WT). There were also significant differences in IgM/IgG binding between GTKO and TKO pigs (**P < 0.01; ***P < 0.001). **c**, **d** Human IgM/IgG binding of AECs was significantly reduced in all transgenic pigs compared to that in WT (***P < 0.001 vs. WT). However, there was no further reduction in IgM and IgG binding in TKO pigs when compared to that in GTKO pigs. **e**, **f** Human IgM/IgG binding of CECs in GTKO and TKO pigs was markedly reduced when compared to that in WT pigs (***P < 0.001 vs. WT), but there were no significant differences in IgM/IgG binding between GTKO and TKO pigs. Experiments were performed in quadruplicate.

To investigate the immunoreactivity of pig-to-human xenotransplantation, we compared the IgM and IgG binding of human antibodies in the PBMCs, AECs, and CECs isolated from GTKO, TKO, and WT pigs (Fig. 7). IgM and IgG binding to the PBMCs of GTKO and TKO pigs were significantly reduced when compared to that of the WT (Fig. 7a, b, ***P < 0.001). IgM and IgG binding to PBMCs from TKO pigs were significantly lower than that in GTKO pigs (**P < 0.01, ***P < 0.001). IgM and IgG binding to AECs and CECs in all transgenic pigs was significantly decreased when compared to WT pigs (Fig. 7c, d, e, f, ***P < 0.001). However, there were no differences in IgM or IgG binding to AECs and CECs between transgenic pigs.

### Human complement-dependent cytotoxicity

**Fig. 8:**
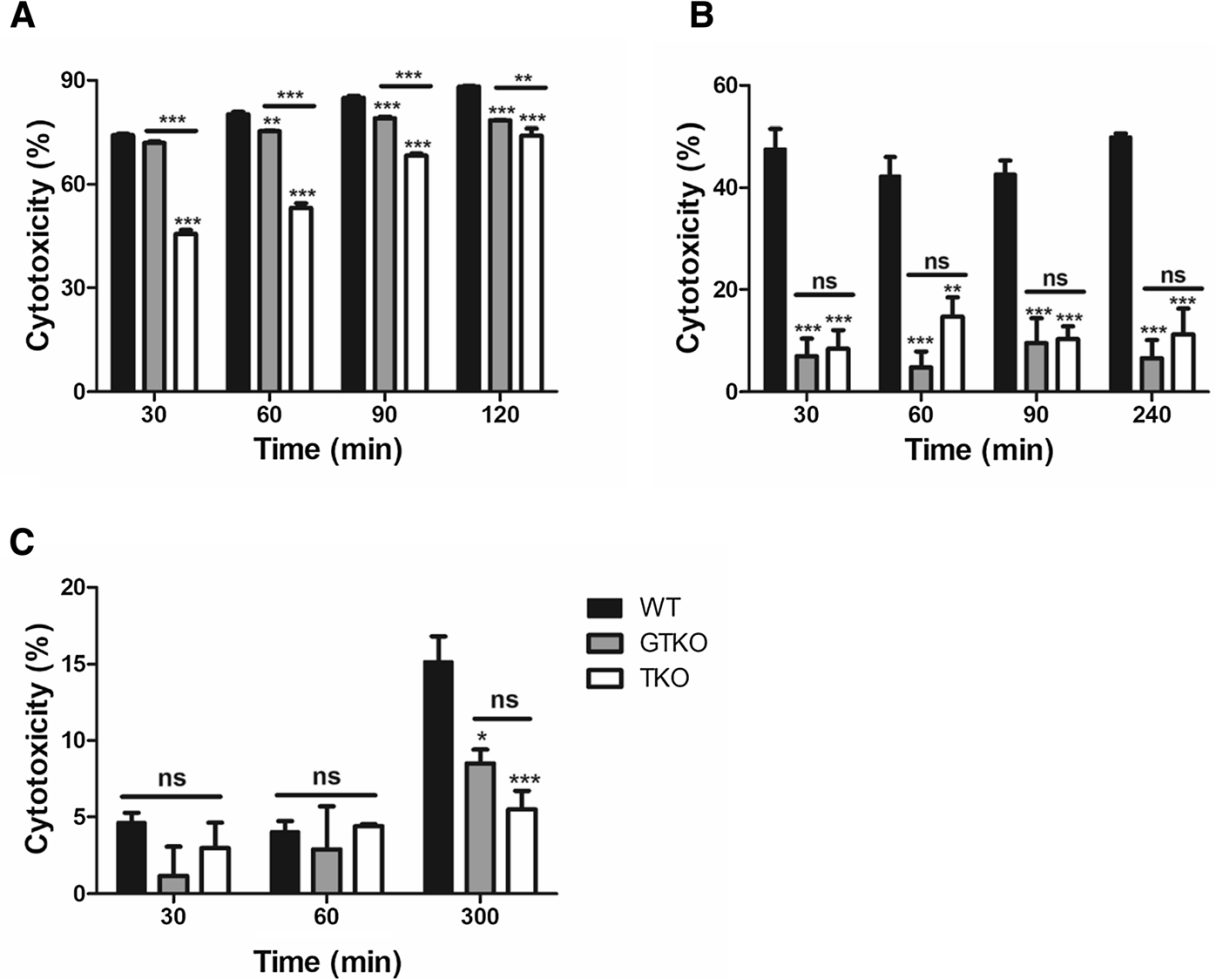
Cytotoxicity of PBMCs, AECs, and CECs from GTKO, TKO, and WT pigs. **a** The cytotoxicity of GTKO pigs on PBMCs was significantly lower than that on WT pigs at 60, 90, and 120 min (**P < 0.01; ***P < 0.001 vs. WT), and there was further significant reduction in the cytotoxicity of TKO pig compared to that of GTKO or WT pigs (**P < 0.01; ***P < 0.001) at all time points. **b** The cytotoxicity of transgenic pigs on AECs was significantly decreased compared to that of WT pigs at all time points (**P < 0.01; ***P < 0.001 vs. WT), but there was no difference in cytotoxicity between GTKO and TKO pig AECs. **c** The cytotoxicity of transgenic pigs on CECs was significantly reduced from WT at 300 min (*P < 0.05; ***P < 0.001 vs. WT), but there was similar cytotoxicity between GTKO and TKO pigs. Experiments were performed in quadruplicate.

We assessed the ability of human serum to cause complement-mediated lysis of PBMCs, AECs, and CECs from GTKO, TKO and WT pigs. The pig cells were incubated with 50% pooled normal human complement serum for 300 min. The cytotoxicity of PBMCs from TKO pigs was significantly decreased when compared to that of WT or GTKO pigs at all time points (Fig. 8a, (**P < 0.01, ***P < 0.001 vs. WT or GTKO). The cytotoxicity of PBMCs from GTKO pigs was significantly decreased from that of WT pigs at 60, 90, and 120 min (**P < 0.01, ***P < 0.001 vs. WT). The cytotoxicity of AECs from all transgenic pigs was significantly decreased from that of WT pigs at all time points (Fig. 8b. **P < 0.01, ***P < 0.001 vs. WT). However, there was no difference in the cytotoxicity of AECs from GTKO and TKO pigs at any time point. The cytotoxicity of CECs from transgenic pigs was significantly decreased from that of WT pigs at 300 min (Fig. 8c, *P < 0.05, ***P < 0.001 vs. WT). However, there was no difference in the cytotoxicity of CECs from GTKO and TKO at any time point.

## Discussion

Pig organs and tissues offer a promising solution to the problem of shortages of organs for transplantation into humans. However, because pigs and humans are different species, human antibodies recognize pig cells as xenogenic antigens, thereby triggering the rejection of transplanted tissues (Hryhorowicz et al. 2017). To facilitate the clinical application of xenotransplantation, understanding the antibody-mediated rejection of transplanted organs is essential (Butler et al. 2016b). Researchers have identified several xenoantigens from pigs and have demonstrated that modifications to the genome of donor pigs using genetic engineering techniques such as ZFN, TALEN, and CRISPR/Cas9 are valuable for generating pig organs that have lower immunogenicity (Hryhorowicz et al. 2017; Niemann and Petersen 2016).

In this study, we generated *GGTA1/CMAH/A3GALT2* TKO pigs using CRISPR/Cas9 genome editing and investigated the effect of this triple gene KO. The CRISPR/Cas9 system is known to be highly efficient for editing genes, is easy, relatively straightforward, and rapid, and can be used to target multiple genes simultaneously (Mehravar et al. 2019). Recently, one-step genome editing using intracytoplasmic microinjection of fertilized zygotes with a CRISPR/Cas9 vector has been successful (Petersen et al. 2016). However, direct injection of CRISPR/Cas9 molecules can cause the system to continue to operate at various stages of embryo development, resulting in genetic mosaicism of transgenic animals (Mehravar et al. 2019; Sato et al. 2018). For these reasons, we used the SCNT system to generate GTKO and TKO pigs in this study. Although the cloning efficiencies were less than 2% (Table 2), the piglets that were produced were genetically identical to the donor cells (Fig. 3).

The expression of αGal was barely detectable in all transgenic pigs. Neu5Gc was not expressed in TKO pigs (Fig. 4). Previous studies in other animals have shown that iGb3 levels are species- and tissue-specific. iGb3 is only detected in the thymus and murine dorsal root ganglia of rats and is not detectable in humans, pigs, or other tissues of mice (Christiansen et al. 2008; Speak et al. 2007). However, *A3GALT2* mRNA is ubiquitous within pig tissues and endothelial cells (Puga Yung et al. 2012). Therefore, we confirmed the expression of the A3GALT2 protein in the AECs of TKO pigs using a synthetic polyclonal antibody instead of iGb3 (Fig. 5). We found that the AECs of TKO pigs rarely express A3GALT2 more than GTKO or WT pigs. This result indicated that three targeted genes were disrupted completely by the CRISPR/Cas9 constructs (Figs. 4, 5).

However, there were no significant differences in IB4 binding in tissues from GTKO and TKO pigs (Fig. 4a). We could not detect any differences in IB4 binding at a cellular level between GTKO and TKO pigs (Fig. S3). Similarly, Butler et al. showed that IB4 binding in PBMCs, spleen and lymph node cells did not differ between GTKO and *GGTA1/A3GALT2* DKO pigs and suggested that silencing of the *A3GALT2* gene did not affect IB4 binding (Butler et al. 2016b). Galili et al. demonstrated that because the binding affinity of the lectin and αGal epitope is low, the detection of the relatively small amount of αGal epitope on cell surfaces may be insufficient to allow an observer to draw any conclusions (Galili et al. 1998). Therefore, we further attempted to detect the αGal epitope on the pig cells (PBMCs, AECs and CECs) using αGal epitope monoclonal antibody. However, the level of αGal epitope expression was already close to zero in GTKO pigs, and we could not measure any additional reduction in its expression associated with *A3GALT2* KO (Fig. 6). Shao et al. showed that αGal epitope expression in various organs of *A3GALT2* KO mice decreased from 21.74 to 5.19% compared to WT mice (Shao et al. 2018). It is possible that the *A3GALT2* gene contributes to αGal epitope expression in mice rather than in pigs.

We evaluated human antibody binding and human complement-mediated cytotoxicity of different cell types from the pigs. Human preformed natural antibodies bind to pig organs or tissues during pig-to-human xenotransplantation. Antibody deposition causes complement-mediated injury of the grafts, leading to thrombosis, interstitial hemorrhage, and edema, all of which disrupts graft function (Cooper et al. 2015). We examined the binding potential of the most important human antibodies, namely, IgM and IgG, and cytotoxicity to PBMCs, AECs, and CECs isolated from GTKO, TKO, and WT pigs. The human antibody binding ability of PBMCs, AECs, and CECs from the transgenic pigs was significantly reduced when compared to that of WT pigs, but these results were different in TKO and GTKO pigs depending on the cell type (Fig. 7). In the PBMCs of TKO pigs, the binding of human antibodies was significantly lower than that in GTKO pigs (Fig. 7a, b). The cytotoxicity of PBMCs from TKO pigs was markedly reduced from those of GTKO or WT pigs at all time points (Fig. 8a). As we did not examine *GGTA1/CMAH* DKO pigs, it is not clear whether the reduction in antibody binding and cytotoxicity in the TKO pigs is caused by *CMAH* KO alone or whether there is an additional effect from the *A3GALT2* KO. However, because we could not observe additional reductions in αGal expression in TKO cells when compared with GTKO, we can conclude that these results were due to the *CMAH* gene KO.

The human antibody binding and cytotoxicity of AECs were similar in GTKO and TKO pigs (Fig. 7c, d, and Fig. 8b). Both the antibody binding and cytotoxicity of the AECs were dramatically reduced by just a *GGTA1* single gene KO compared with WT pigs. As AECs were cultured in a medium containing 2% of fetal bovine serum (FBS), we additionally confirmed whether FBS induced false-positive Neu5Gc expression using FACS analysis (Wang et al. 2016). As a result, AECs of TKO pigs were not expressed Neu5Gc differs from GTKO and WT pigs (Fig. S4). The effects of additional gene silencing such as *CMAH* and *A3GALT2* based on GTKO in AECs, unlike PBMCs, do not appear to be significant. PBMCs are used primarily in assays related to the immune reactivity of xenogeneic antigens because they contain a range of immune cells, such as lymphocytes, monocytes, and macrophages (Pourahmad and Salimi 2015). However, it seems that the source of the cells used to demonstrate the effectiveness of xenoantigens *in vitro* should be target organ specific. For studies of solid organ xenotransplantation, porcine AECs have mainly been used to detect humoral immune responses *in vitro*. Zhang et al. suggested that assays based on pig renal microvascular endothelial cells (RMECs) are more useful than AECs to detect immune responses for kidney xenotransplantation because RMECs are more immunogenic than AECs (Zhang et al. 2017a).

In previous reports, GTKO pig organs increased the survival of pig-to-primate transplants, but graft failure did eventually occur (Kuwaki et al. 2005; Yamada et al. 2005). This observation suggests that non-Gal antigens contribute to acute vascular rejection responses (Ezzelarab et al. 2005; Kwon et al. 2013). Many researchers have shown that both *GGTA1* and *CMAH* DKO pigs could reduce the humoral rejection response to xenotransplantation more than GTKO pigs using pig PBMCs, AECs, and RBCs (Hara et al. 2008; Lee et al. 2016a; Lee et al. 2016b; Lutz et al. 2013). It is possible that our results using AECs were different because we used different pig breeds. Previous reports were based on the use of cross-breed pigs, such as Landrace, Yorkshire, Chester white, Large White, or Duroc. However, we used Yucatan miniature pigs. Actually, we compared human antibody binding on PBMCs between commercial cross-breed (Landrace/Yokshire/Duroc) and WT Yucatan miniature pigs. IgM binding was similar between the two groups, but IgG binding in Yucatan miniature pigs was lower than that in cross-breed pigs (commercial cross-breeds vs. Yucatan, IgM 566: 431, IgG 2,564: 1,567, n = 3). This result indicates that Yucatan miniature pigs have lower non-gal antigen than cross-breed pigs, and xenoantigens might differ depending on the breed of pig.

Previous studies reported that the humoral and cellular responses to GTKO, GTKO*/hCD46*, or DKO (*GGTA1/CMAH)/hCD46* pig CECs were dramatically reduced compared with WT pig CECs, but there were no significant differences among the CECs of transgenic pigs (Hara et al. 2011; Lee et al. 2016a; Lee et al. 2016b). As in previous reports, the level of human IgM/IgG binding in CECs was similar between GTKO and TKO transgenic pigs (Fig. 7c). Furthermore, the cytotoxicity of CECs was significantly reduced in transgenic pigs over WT at 300 min, and there was no difference between GTKO and TKO pigs (Fig. 8c). Cornea is an immune-privileged tissue (Hori et al. 2019; Yoon et al. 2021), and CECs appear to have less immunogenicity than PBMCs or AECs. These results appear to indicate that a *GGTA1* single gene knockout is sufficient for corneal xenotransplantation.

Collectively, our data suggest that the genetic modification of donor pigs for xenotransplantation should differ depending on the target organ and silencing of additional genes such as *CMAH* or *A3GALT2* based on GTKO might not be essential in Yucatan miniature pigs. However, our study has limitations due to the use of *in vitro* testing. Further study should focus on the characterization of Yucatan miniature pigs and the effects of genetically modified pig-to-nonhuman primate organ transplantation. Although *A3GALT2* KO pigs rarely had an effect on pig-to-human immune reactivity in our study, it could provide more information on the role of iGb3 on NKT cell activity in preclinical test. Recently, preclinical studies have reported that pigs with a *CMAH* gene knocked out express new xenoantigens called “forth xenoantigen” in pig-to-Old World NHP organ transplantation (Cooper et al. 2020; Cui et al. 2020; Yamamoto et al. 2020). This finding indicates that it is, as yet, almost impossible to confirm the effect of *CMAH* KO in preclinical tests. If the deletion of the *CMAH* gene is not needed in Yucatan miniature pigs, they could be an ideal model animal for the study of xenotransplantation.

## Supplementary Information

Below is the link to the electronic supplementary material.

Supplementary file 1 (DOCX 3774 KB)

## Data Availability

All data generated or analyzed during this study are included in this published article and its supplementary information files.
