# Supplementary material for: Human immune reactivity of GGTA1/CMAH/A3GALT2 triple knockout Yucatan miniature pigs
Source: Transgenic Res. 2021 Jul 7;30(5):619–34. doi: 10.1007/s11248-021-00271-w (PMC8478729; doi:10.1007/s11248-021-00271-w)
Supplement: Supplementary file 1 — (DOCX 3774 KB) [file 11248_2021_271_MOESM1_ESM.docx]

**SUPPLEMENTARY DATA**

**Table S1** Primer sets used for CRISPR/Cas9-mediated gene targeting

| Gene | Sequences (5′-3′) | PCR product |
| --- | --- | --- |
| *GGTA1* | F: AGAATCACCAGTCAGGTAAGCCACTCC | 496 |
|  | R: TTGGAAGACCTGATCCACGTCCATGCAG |  |
| *CMAH* | F: TGTTCTACTTCTGCATCACTC | 328 |
|  | R: CAGCTAAATCACTCATTCAGC |  |
| *A3GALT2* | F: GACAGCAGAGCAGCACTTCAT | 344 |
|  | R: TGTCACGCTCAAAGGGCAGCA |  |

**
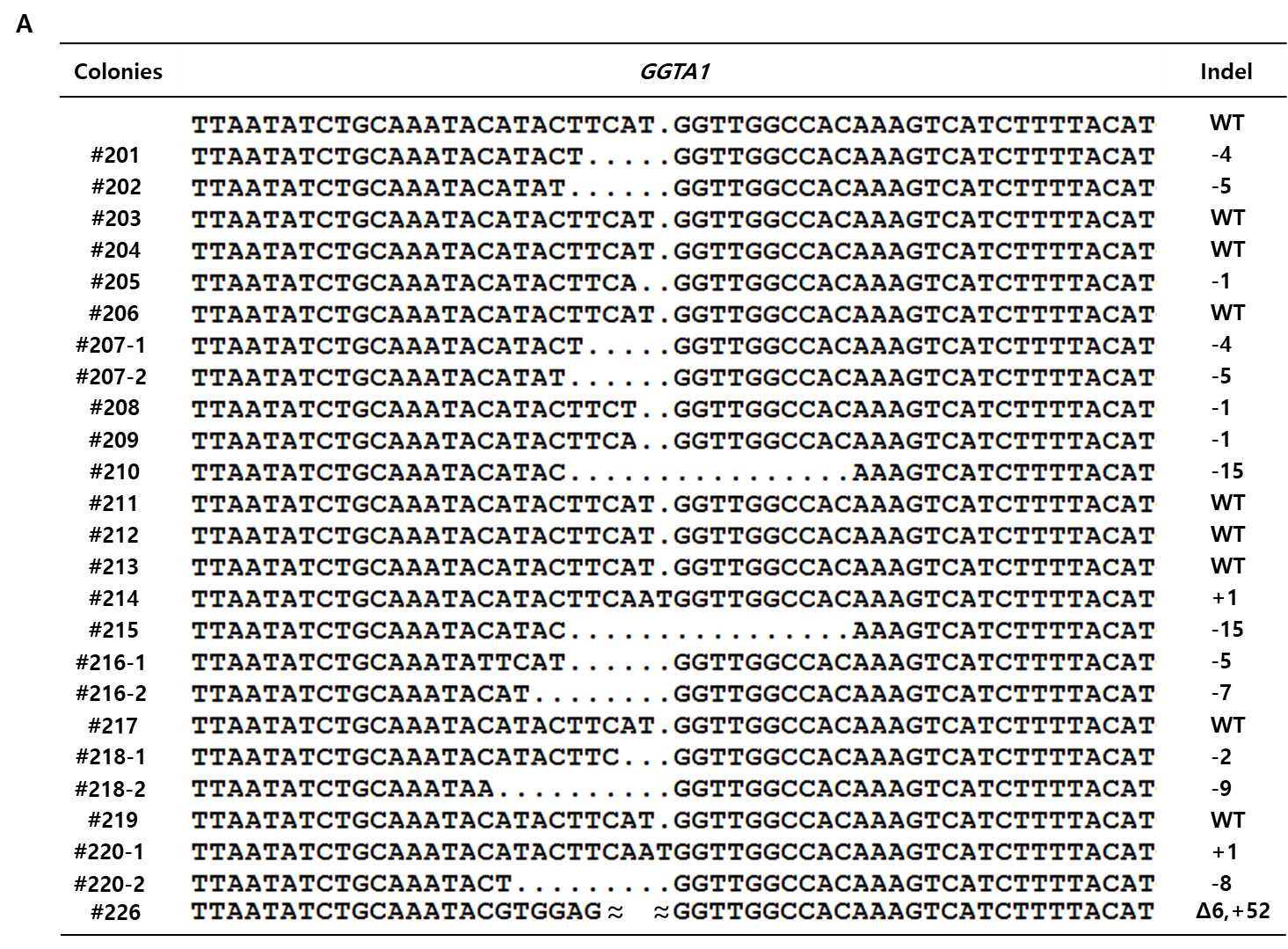
**

**
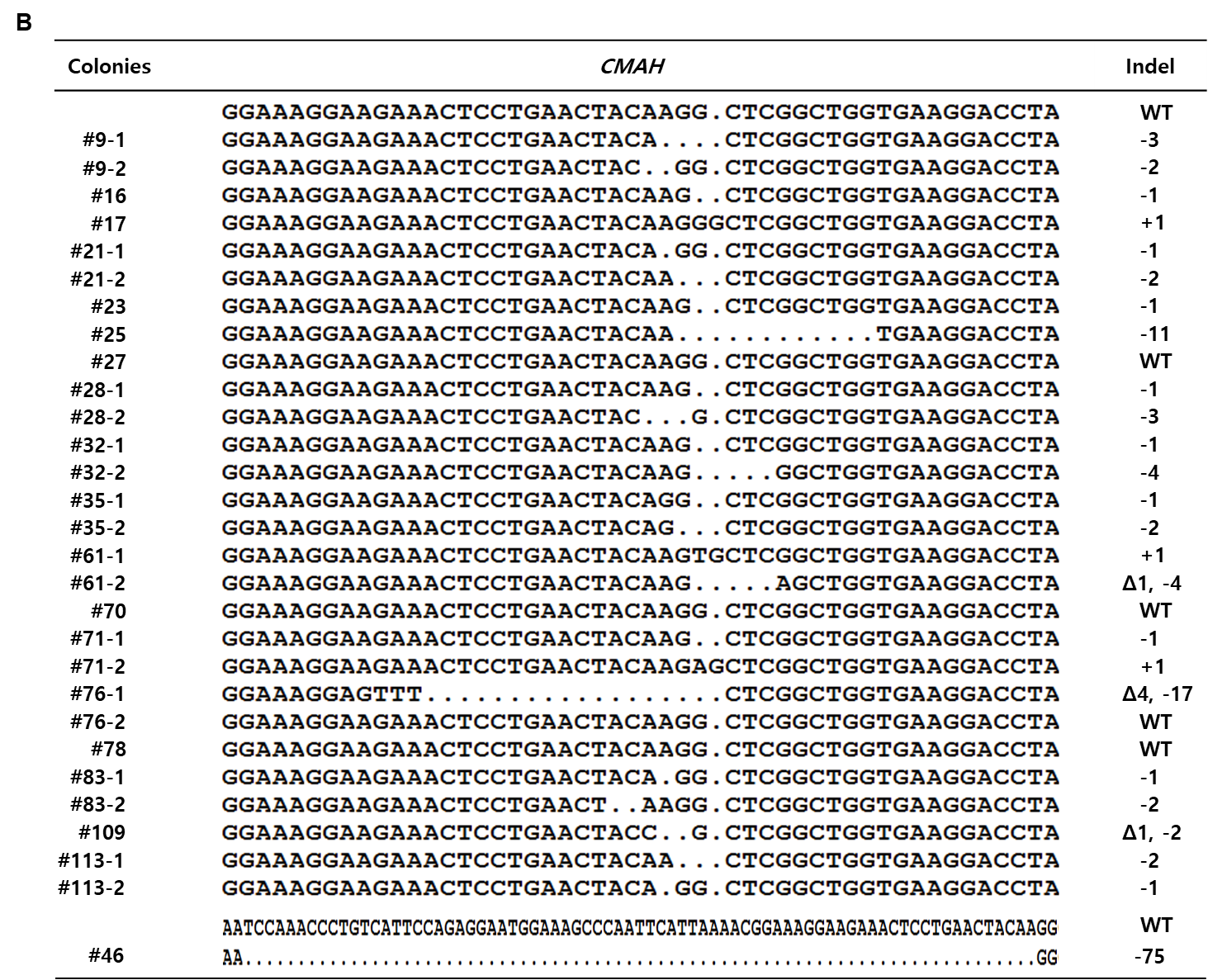
**

**
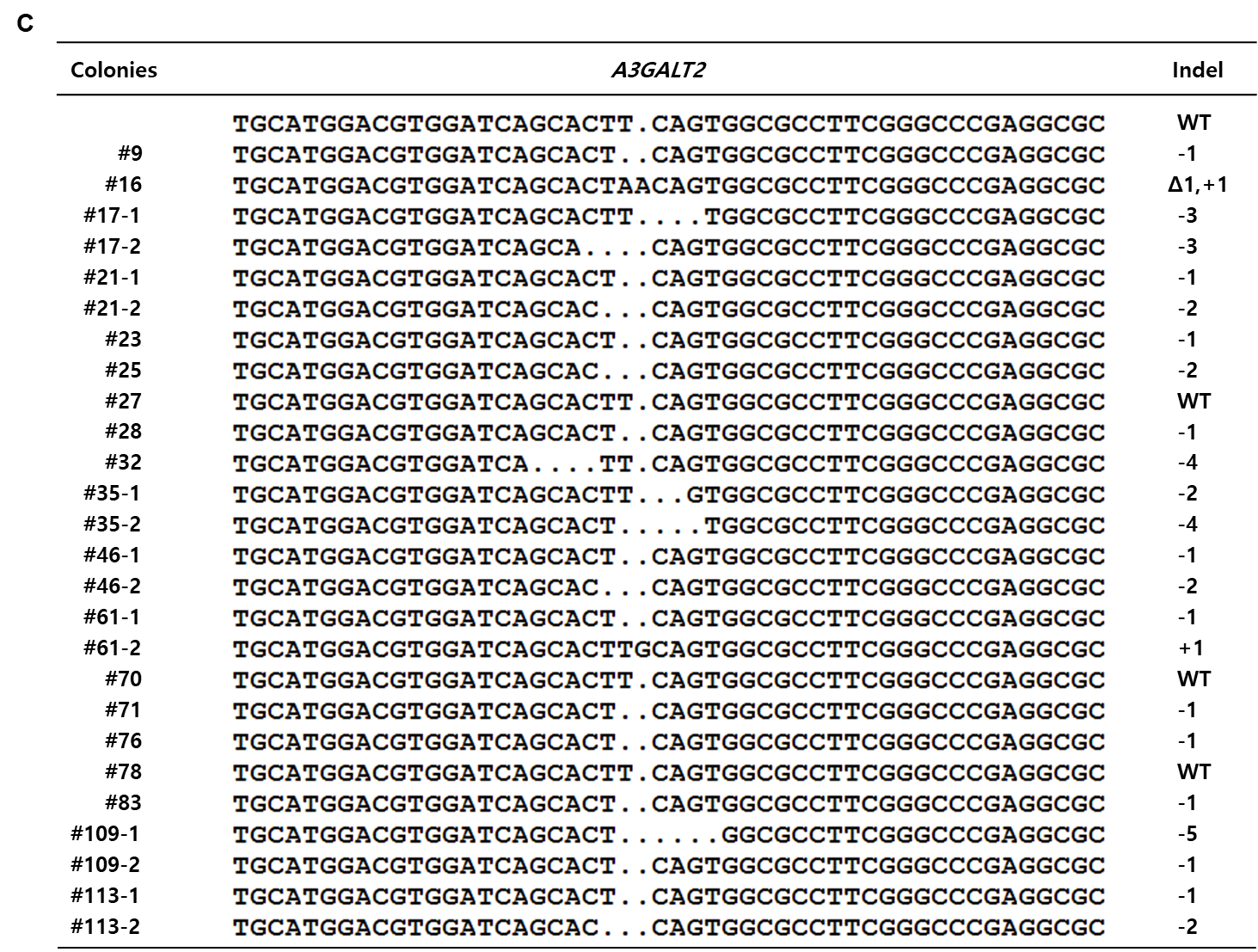
**

**Figure S1** CRISPR/Cas9-mediated mutation of cell colonies. **a** Sequencing results of *GGTA1* **b** *CMAH* **c** *A3GALT2* cell colonies

**
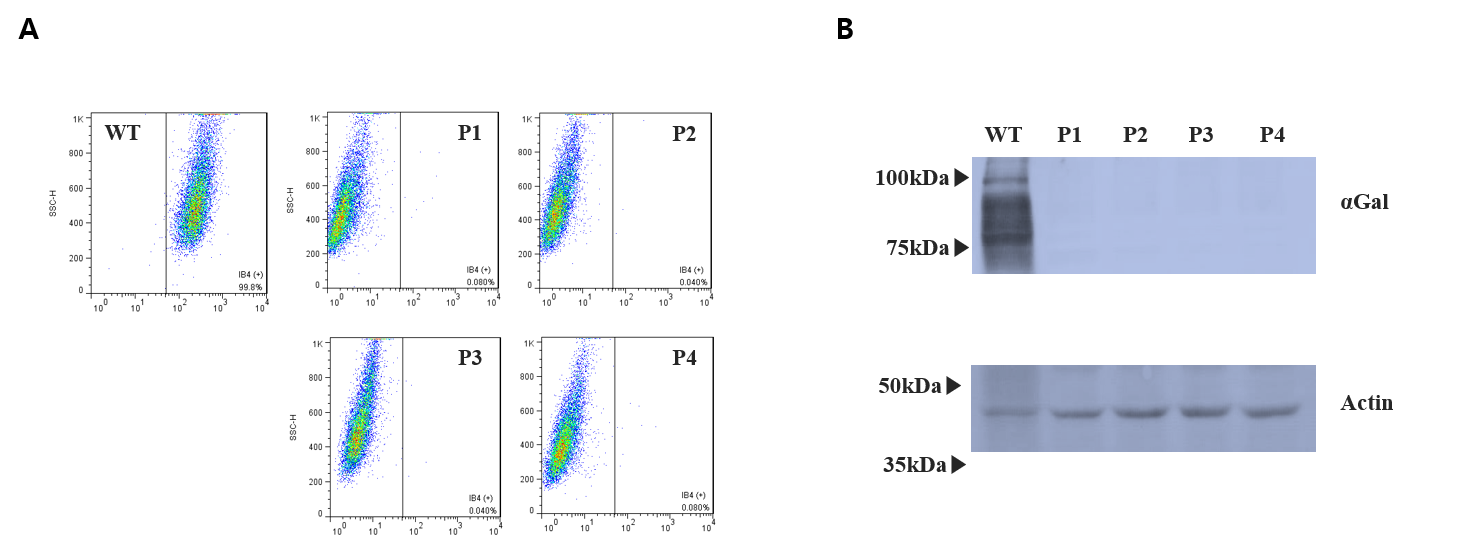
**

**Figure S2** Absence of αGal expression in four GTKO piglets. **a** Flow cytometry analysis of the ear fibroblasts indicated the absence of the αGal antigen in the GTKO piglets. The ear fibroblasts were stained by GS-IB4 lectin. **b** Western blot assay in four piglets showing successful GGTA1 gene knockout. WT; wild-type, P1-P4; GTKO piglets 1-4.


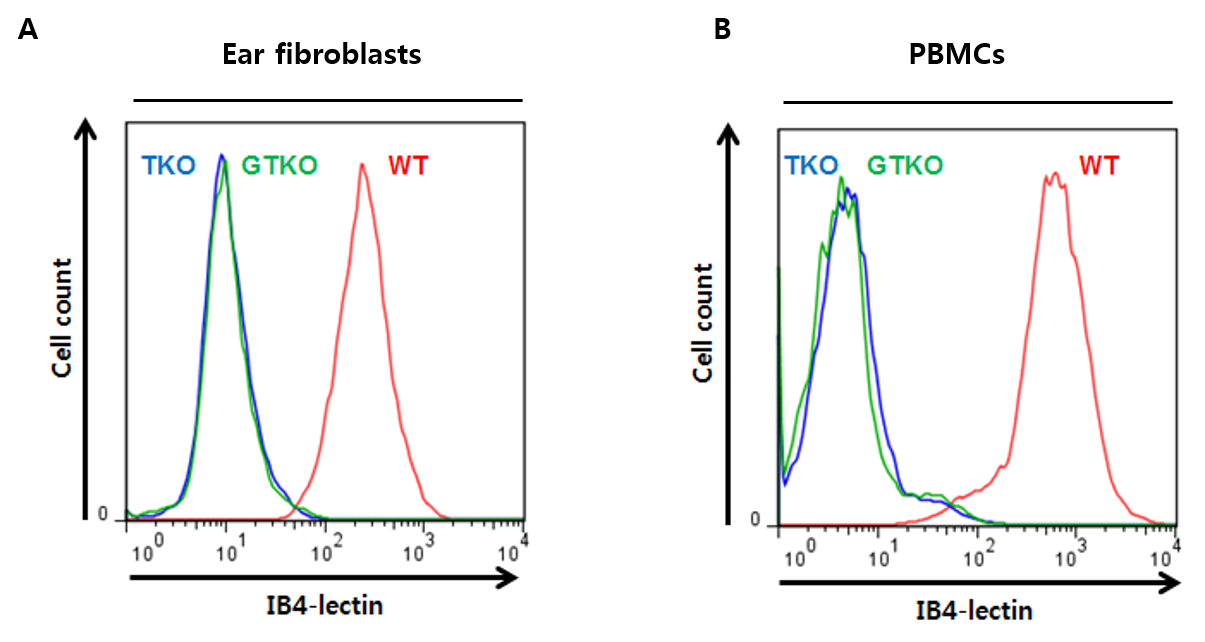


**Figure S3** IB4 binding of GTKO, TKO, and WT pig cells. **a** Ear fibroblast cells **b** PBMCs. IB4 binding did not differ between GTKO and TKO pigs (WT, red; GTKO, green; TKO, blue).


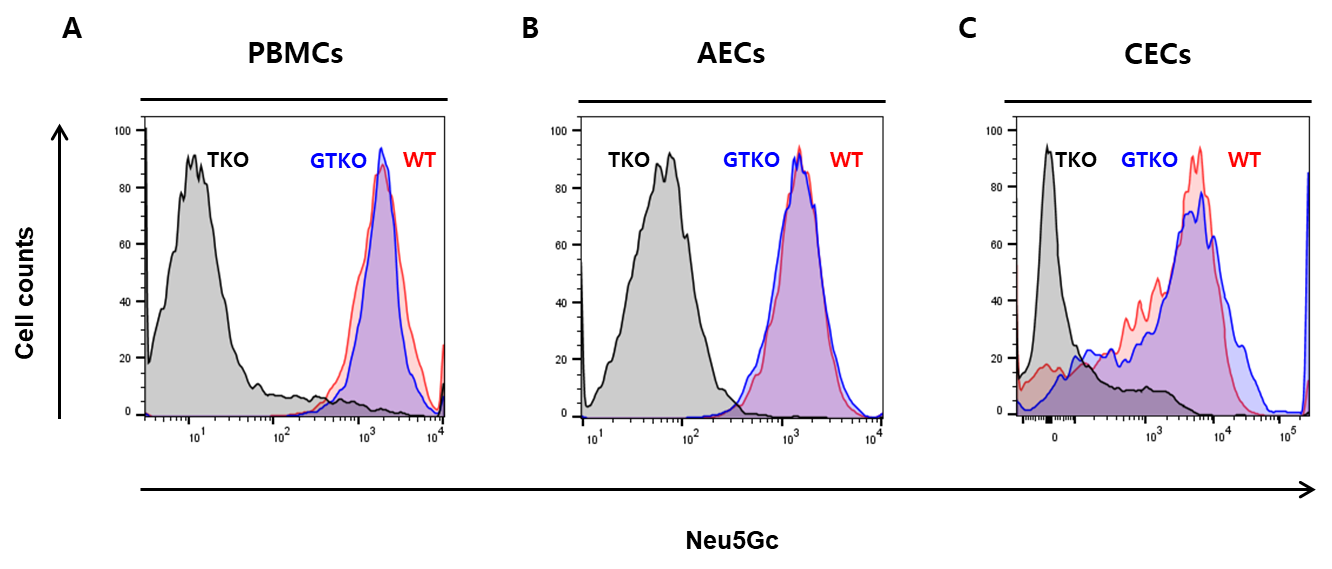


**Figure S4** Neu5Gc expression on various pig cells. **a** PBMCs **b** AECs **c** CECs. Neu5Gc expression was confirmed in all types of cells from GTKO and WT pigs except TKO pig. (WT, red; GTKO, blue; TKO, black).
